# Influence of Dental Implant Diameter and Bone Quality on the Biomechanics of Single-Crown Restoration. A Finite Element Analysis

**DOI:** 10.3390/dj9090103

**Published:** 2021-09-06

**Authors:** Eduardo Anitua, Naiara Larrazabal Saez de Ibarra, Iñigo Morales Martín, Luis Saracho Rotaeche

**Affiliations:** BTI Biotechnology Institute, Jacinto Quincoces, 39, 01007 Vitoria, Spain; naiara.larrazabal@bti-implant.es (N.L.S.d.I.); inigo.morales@bti-implant.es (I.M.M.); luis.saracho@bti-implant.es (L.S.R.)

**Keywords:** biomechanics, finite element analysis, single-unit implant, short dental implant

## Abstract

Background: Success of an implant-supported prosthesis is highly dependent on implant diameter and bone quality. The objective of this study is to assess these two variables under axial or 30° angulated loading. Methods: The study was conducted using finite element model simulations of dental implants with an unchanging length of 6.5 mm and varying diameters of Ø3.3; Ø3.5; Ø3.75; Ø4, Ø4.25 and Ø4.75 mm. The implants were placed in an axial position and a 2 mm high straight transepithelial (intermediate abutment) was used to perform a single tooth restoration. Four bone quality scenarios, Type IV, III, II or 0-I bone, were simulated from a simplified model of the mandible. A 200N load was applied both axially and at a 30° angle to the occlusal surface of the prosthesis, which was 11 mm above the implant platform, and the equivalent Von Mises stress in the bone was analyzed. Results: The maximum stress value was obtained for the Ø3.3 implant in Type IV bone (235 MPa), while the lowest value was obtained for the Ø4.75 implant and in Type 0-I bone (41 MPa). Regardless of the implant diameter, an improvement in bone quality produced a reduction in bone stress. The same effect was observed as the implant diameter was increased, being this effect even more pronounced. Conclusions: Implant diameter has an important effect on bone stress, with a reduction in stress as the implant diameter increases.

## 1. Introduction

Finite element analysis is a tool that are used to simulate the stress in the implant system and also the bone contacting the implant. During function, axial forces and bending moments are transmitted to the dental implant and thus to the surrounding bone. The stability of the dental implant and the supporting tissue would be affected by the manner in which these mechanical stresses are transmitted to bone. Several factors are affecting stress transmission to bone. [1] Among them are the type of loading, the union of the implant to bone, the dimensions of the implants, the implant surface, the type of the prosthesis, and the characteristics of the hosting bone. [1,2] For example, the dental implant design may cause excessively high or low stresses in peri-implant bone affecting its stability [1,3].

The success of dental implant is sensible to the stresses that are transferred to the supporting bone [1]. Implant diameter and bone quality are two major factors that influence the biomechanics of an implant-supported prosthesis [1,2]. In a previous study using finite element modeling Anitua et al. already highlighted the important effect of the implant diameter on the biomechanics of the prostheses, and concluded that the use of wider implants may be an option to reduce the stress on the bone close to the implant [4]. Nevertheless, this study did not consider different bone quality scenarios, and all studies were carried out on a Type 0-I bone. 

A similar study was conducted by Baggi et al., who observed that the maximum stress was found at the neck of the implant. They also observed in their study that both maxi mum stress values and regions of stress concentration in cortical bone decreased as implant diameter increased, while more favorable stress distributions were observed in trabecular bone as implant length increased [5].

The reduction in the stress in bone has been significantly higher by increasing the diameter than by increasing the length of the implant [6]. Bone stress is mainly concentrated in the cortical bone independently of the implant design and, therefore, a longer implant is not effective to counteract the effect of the crown size [7,8]. Eazhil et al. reported findings in this regard using finite element models, and observed how the equivalent Von Mises stress is concentrated in the bone close to the collar of the implant [9]. They also identified implant diameter as a more effective design parameter than implant length, and found that a wider implant dissipated stress better than a longer one.

The implant treatment success is also significantly influenced by the bone quality of the placement site and the primary stability achieved [10].

According to Rabel et al., primary implant stability is the combination of the quantity and quality of the recipient bone, the implant design and the employed surgical technique [11]. Azcarate-Velázquez et al. carried out a finite element modeling based study to assess the effect of bone type on the bone stress under compressive and oblique loading in two types of dental implants. [12] In their study, they observed that a reduction in bone quality resulted in worse stress distribution and an overloaded cortical bone. In their study, they also observed that under oblique loading, stress in the cortical region was significantly higher than under compressive loading.

Several studies have assessed short implants as a single-unit implant in posterior regions [13,14]. However, Mezzomo et al. in a meta-analysis have found that implant length ≤ 8 mm increased the marginal bone loss, mean implant failure proportion and biological complications [15]. Recent data from a meta-analysis indicates that C/I ratio of single-tooth and nonsplinted impants did not increase the occurrence of biological or technical adverse events [16]. Guljé et al. have observed that high C/I ratio (2.14 ± 0.42) of 6-mm long implants (single-unit implants) has not been associated with marginal bone loss or technical complications [17]. Clinical and biomechanical studies will highlight factors that may influence the success of single-unit restorations supported by short implants. The present study aims to evaluate the effect of the implant diameter on stress transmission to the bone, also introducing the variable of the different types of bone quality. Thus, a more accurate simulation of the different possible clinical situations can be achieved.

## 2. Material and Methods

**Figure 1:**
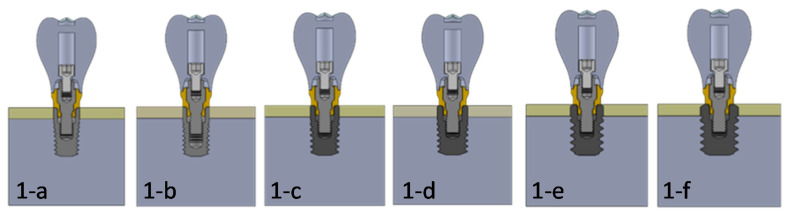
(**1-a**). 3D model with an Ø3.3 implant in axial position and Type III bone. (**1-b**). 3D model with an Ø3.5 implant in axial position and Type III bone. (**1-c**). 3D model with an Ø3.75 implant in axial position and Type III bone. (**1-d**). 3D model with Ø4 implant in axial position and Type III bone. (**1-e**). 3D model with an Ø4.25 implant in axial position and Type III bone. (**1-f**). 3D model with Ø4.75 implant in axial position and Type III bone.

A simplified jaw model consisting of a cylinder of diameter Ø15.5 mm and height H15.5 mm was used for the calculations (Figure 1(1-a–1-f)). 

The definition of the different scenarios according to bone quality was based on Anitua et al. classification of bone types [2,10]. It is a modification of the classification proposed by Lekholm and Zarb. [18] The applied classification was based on bone density measured in Hounsfield units and thickness of the cortical bone with the aid of computer software (BTI Scan, BTI Biotechnology, Vitoria, Spain); defining 6 bone types 0, I, II, II, III, IV and V.

**Figure 2:**
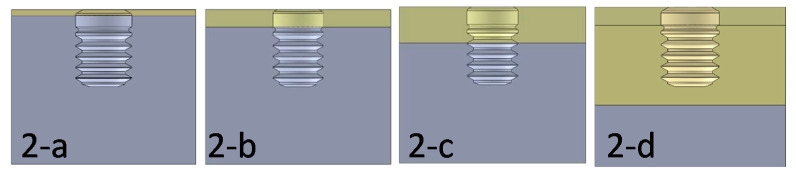
(**2-a**). Scenario 1, Type IV bone. (**2-b**). Scenario 2, Type III bone. (**2-c**). Scenario 3, Type II bone. (**2-d**). Scenario 4, Type 0-I bone.

Depending on the type of bone tissue, the following cases were simulated:

**Case 1** (Figure 2(2-a)): simulates a Type IV bone (bone density of 400 to <500 U) with a 0.5-mm thick cortical bone surrounding the cancellous bone [2,10]. 

**Case 2** (Figure 2(2-b)): simulates a Type III bone (Bone density of 550 to <850 U) with a 1.5-mm thick cortical bone surrounding the cancellous bone [2,10].

**Case 3** (Figure 2(2-c)): simulates a Type II bone with a 3-mm thick cortical bone surrounding the cancellous bone. Bone density of 850 to <1000 HU [2,10].

**Case 4** (Figure 2(2-d)): simulates a Type 0-I bone with an 8-mm thick cortical bone surrounding the cancellous bone. Bone density greater than 1000 HU [2,10].

In all cases, the implant was considered as fully osseointegrated. For the generation of the models, the geometry of the threads of all the components was simplified, generating threads with revolution geometry, instead of helical geometry, in order to be able to apply the symmetry constraint to the model. As this is a comparative study, this simplification in the geometry of the models allows a more reliable comparison between the different case studies by preventing a small geometric detail from biasing the results in excess.

**Figure 3:**
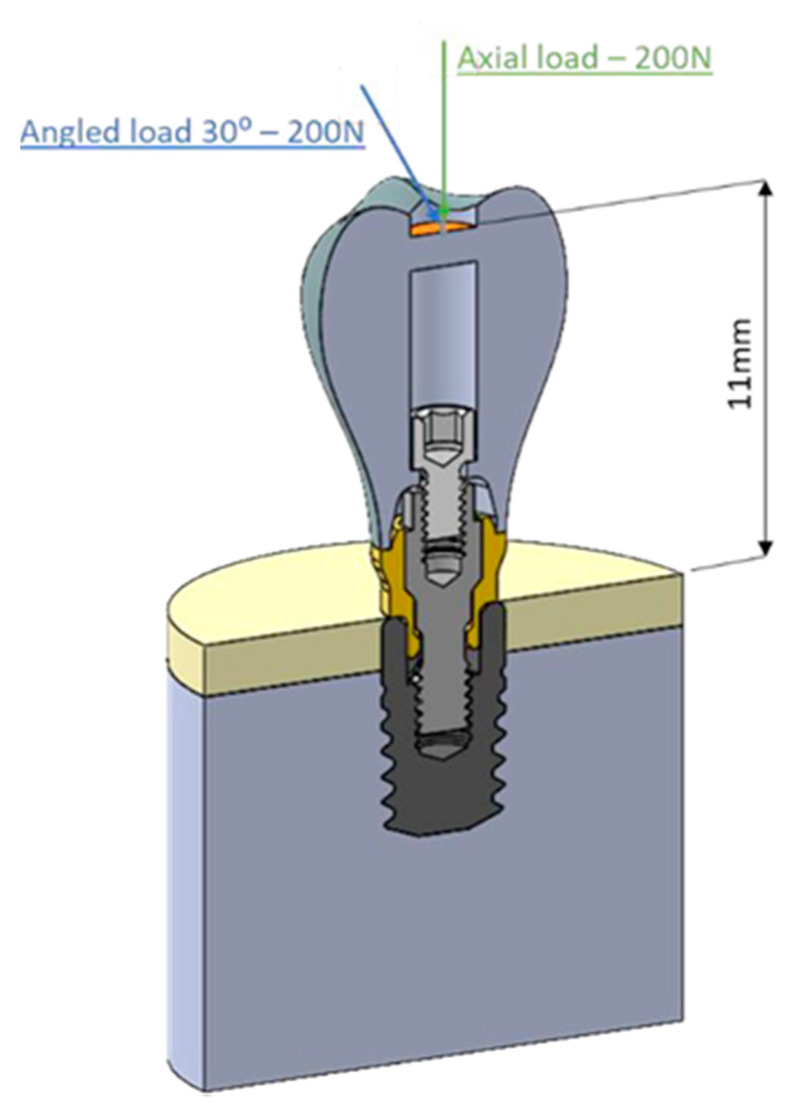
In orange, load application area. In green, the axial load of 200N, in blue, an angled load at 30° of 200N.

The prosthesis was modeled in order to ensure in all models a distance of 12 mm from the top of the prosthesis to the implant platform. As the load was applied in a plane located 1 mm below the prosthesis cusps, the distance between the load application plane and the implant platform resulted to be 11 mm (Figure 3).

The implants used in the models were BTI “Core” narrow internal connection (BTI Biotechnology Institute, Vitoria, Spain) with diameters Ø3.3; Ø3.5; Ø3.75; Ø4; Ø4.25 and Ø4.75 and 6.5 mm in length. The mechanical properties used in each of the components are detailed in Table 1. In all models, a 2-mm high Multi-im transepithelial –intermediate abutment- (BTI Biotechnology Institute, Vitoria, Spain) was used.

**Figure 4:**
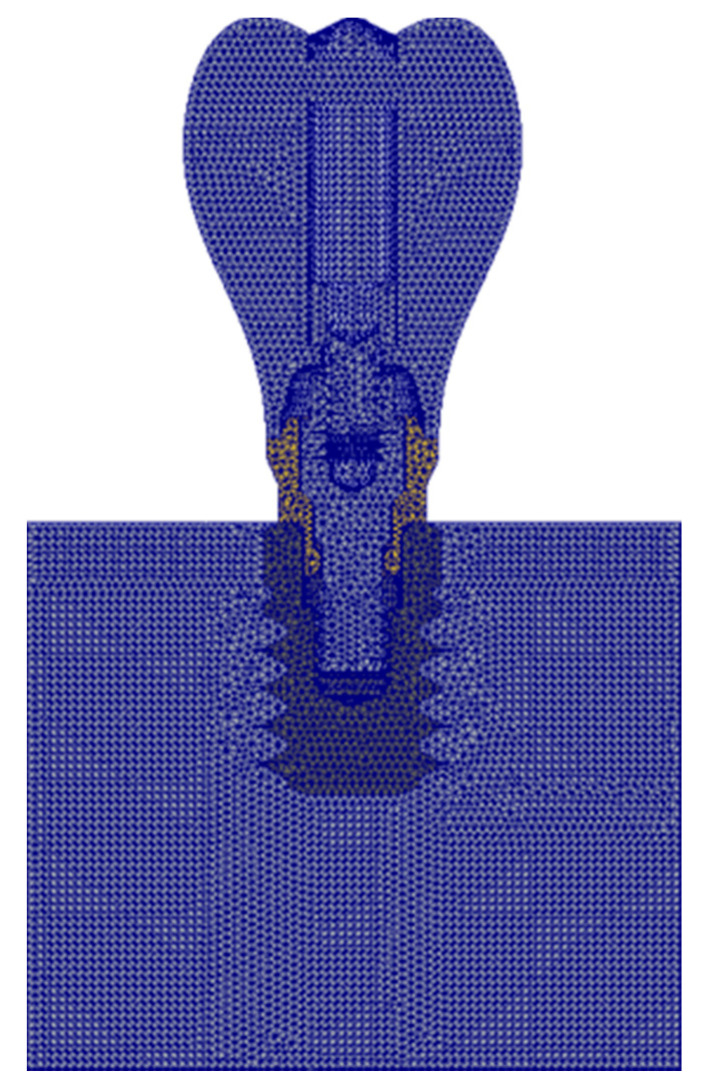
Finite element model mesh.

For the 3D modeling of the parts, the CAD design software Solidworks Professional 2020 (Dassault Systèmes^®^, Vélizy-Villacoublay, France) was used, based on the manufacturing specifications of the components. The calculation software Solidworks Simulation Premium 2020 (Dassault Systèmes^®^, Vélizy-Villacoublay, France) was used to generate the finite element model. For the meshing, 10-node tetrahedral elements with a maximum element size of 0.2 mm were used (Figure 4). An element size below 0.3 mm has been shown to be valid for modeling the bone-implant interface [19].

All materials were modeled with homogeneous, isotropic, and linear properties. The material properties for each of the components are described in Table 1. In all cases, a complete osseo-integration of the implant was assumed, and the contacts between the different components were modeled by means of rigid joints with compatible meshing between the components. All degrees of freedom were restricted on the outer faces of the bone block and the symmetry condition was applied to the model. The axial or 30° angled load of 200 N [20,21] was applied in a uniformly distributed manner on the upper central face of the prosthesis, located at a distance of 11 mm from the bone (Figure 4).

**Table 1:** Material properties of the components used in the finite element models.

COMPONENT	MATERIAL	ELASTIC MODULUS (MPa)	POISSON COEF.
**Dental Implant**	Commercially pure titanium [22]	105,000	0.37
**Prosthesis screw**	Titanium alloy [22]	113,800	0.342
**Transepithelial bodyl**	Commercially pure titanium [22]	105,000	0.37
**Transepithelial screw**	Titanium alloy [22]	113,800	0.342
**Prosthesis**	Co-Cr alloy [20]	218,000	0.33
**N/A**	Cortical bone [20]	13,700	0.28
**N/A**	Cancellous bone [20]	1370	0.3

## 3. Results

After the analysis of the implant performance in the 4 selected bone types and the two loading situations, the maximum values of the equivalent Von Mises stress in the bone was obtained, as well as its distribution in the surrounding bone.

Table 2 shows the maximum values of Von Mises equivalent stress in bone tissue under an axial load of 200N, and Table 3 shows the values for 30° angled loading. It could be observed that the values obtained for the implants under a 30° angled load were significantly higher than in the cases in which the applied load was axial.

Overall, an improvement in bone quality resulted in a reduction of stress in the bone. This reduction in stress ranged from 48-66% for axially loaded implants and 23-40% for oblique loaded implants. It was also observed that the diameter of the implant had an important effect on the distribution of stress in the bone. For the same type of bone, when comparing the smaller diameter implant with the larger diameter implant, bone stress in the former was 4 times higher.

**Figure 5:**
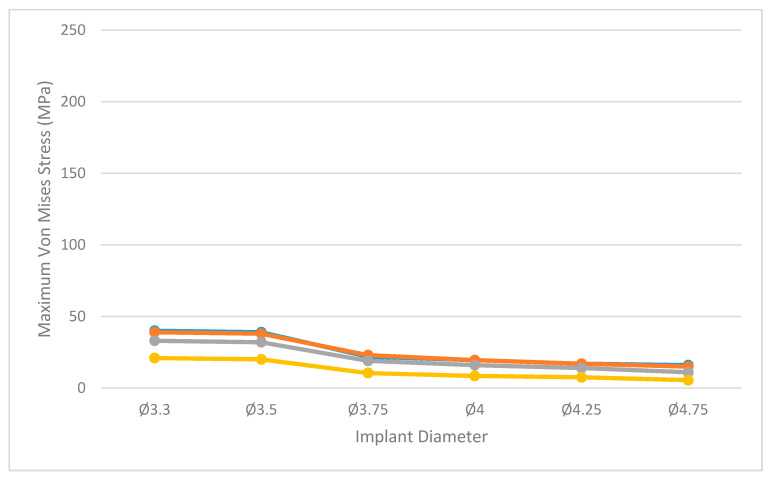
Maximum values of equivalent Von Mises stress (MPa) in bone tissue for each of the study scenarios under an axial load of 200 N. Bone type 0- I (yellow), bone type II (grey), bone type III (orange) and bone type IV (blue).

**Figure 6:**
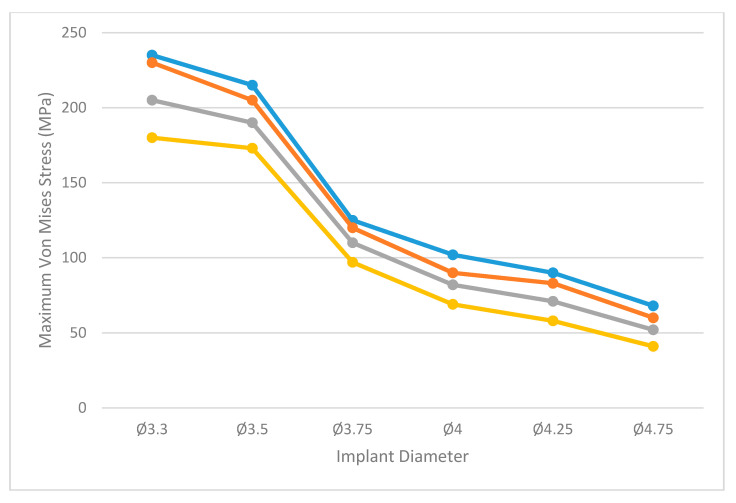
Maximum values of equivalent Von Mises stress (MPa) in bone tissue for each of the study scenarios under a 30° angled load of 200N. Bone type 0- I (yellow), bone type II (grey), bone type III (orange) and bone type IV (blue).

Figure 5 shows the maximum value of the Von Mises stress obtained in each analyzed scenario under axial loading, while Figure 6 shows the values obtained under angled loading. 

**Figure 7:**
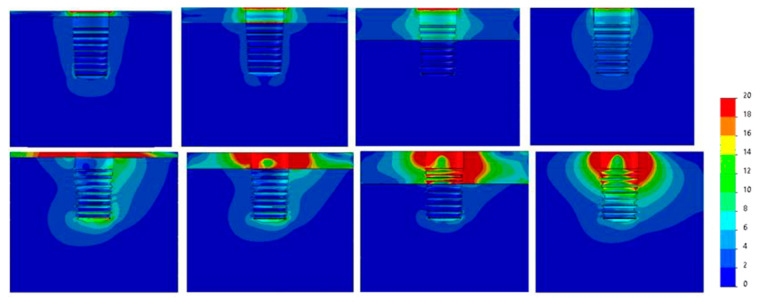
Von Mises equivalent stress (MPa) for Ø3.3 implant cases. Above, axial load, below, angled load. From left to right, Type IV, III, II, 0-I bone.

**Figure 8:**
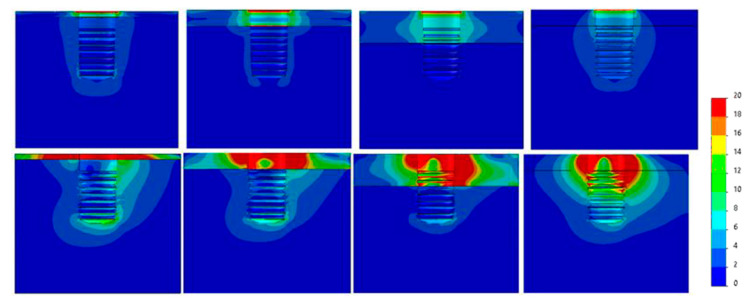
Von Mises equivalent stress (MPa) for Ø3.5 implant cases. Above, axial load, below, angled load. From left to right, Type IV, III, II, 0-I bone.

**Figure 9:**
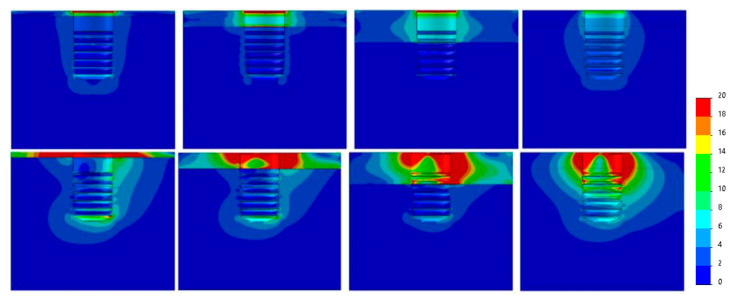
Von Mises equivalent stress (MPa) for Ø3.75 implant cases. Above, axial load, below, angled load. From left to right, Type IV, III, II, 0-I bone.

**Figure 10:**
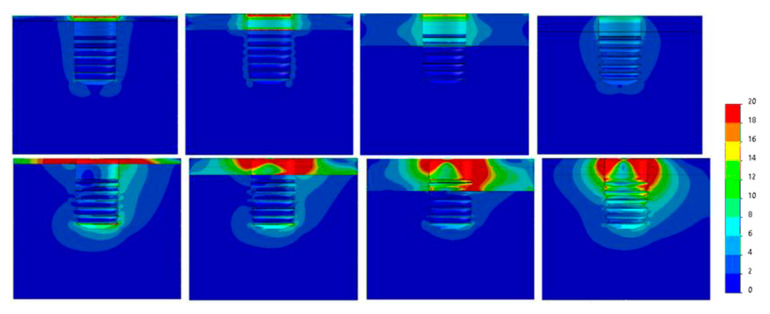
Von Mises equivalent stress (MPa) for Ø4 implant cases. Above, axial load, below, angled load. From left to right, Type IV, III, II, 0-I bone.

**Figure 11:**
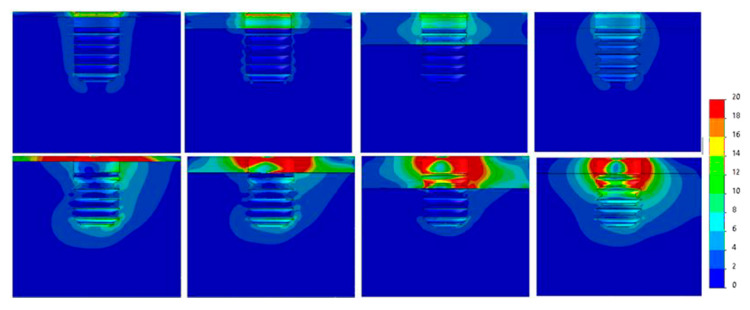
Von Mises equivalent stress (MPa) for Ø4.25 implant cases. Above, axial load, below, angled load. From left to right, Type IV, III, II, 0-I bone.

**Figure 12:**
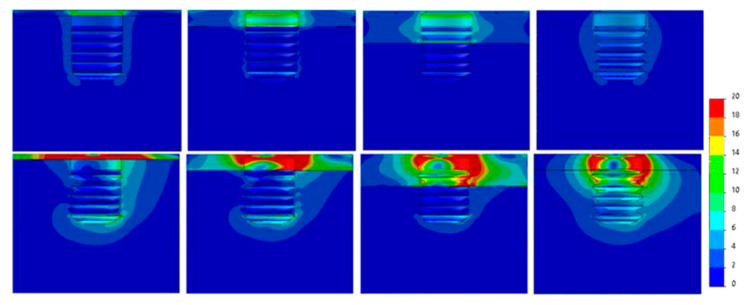
Von Mises equivalent stress (MPa) for Ø4.75 implant cases. Above, axial load, below, angled load. From left to right, Type IV, III, II, 0-I bone.

Figure 7, Figure 8, Figure 9, Figure 10, Figure 11 and Figure 12 show the Von Mises stress distribution in bone for each individual model tested.

## 4. Discussion

The success of a treatment with dental implants is highly dependent on stresses transmission to the supporting bone. This load transfer depends on the factors such as type of loading, the dimensions of the implant and the quality of bone among other factors. Finite element analysis is a tool that are used to simulate the stress in the implant system and also the bone contacting the implant [1].

Under axial loading, the maximum value of bone stress was 40 MPa and it was obtained in the Type IV bone scenario with the narrowest implant (Ø3.3). In contrast, the lowest bone stress was obtained in the scenario of higher density bone and wider implant (Ø4.75). An increase in implant diameter consistently resulted in a reduction of bone stress; this same stress reduction effect was observed as bone quality improved. Similar results were obtained in their finite modeling study by Azcarate-Velázquez et al. [12]. It should be noted, however, that under axial load, no differences in bone stress were observed between bone type IV and III scenarios, whereas among the other bone types the stress reduction effect was clearly observed. 

It can be observed from the obtained results that the region experiencing the highest stress is surrounding the collar of the implant. This effect was also observed by Eazhil et al. in their study [9].

Under oblique loading, the maximum stress value (235 MPa) was obtained with the Ø3.3 diameter implant in the worst bone quality scenario (Type IV). As the implant diameter increased, a reduction in the stress was observed in the bone. The minimum values (41 MPa) were observed for the Ø4.75 implant in Type 0-I bone. These results are in accordance with those obtained in previous studies [5,9]. The former concluded that the use of larger diameter implants could help to better dissipate the acting forces and therefore reduce the stress on the peri-implant bone.

In the present study, the use of a Ø3.3 diameter implant versus a Ø4.75 diameter implant led to 2.5 to 4.4 times higher stress, depending on the bone type. It has been shown that the implant diameter influenced the stress concentration in the bone close to the implant and that this, in turn, affected the implant survival rate [23]. An increase in implant diameter could result in a reduction of stress in the implant and in the surrounding bone, which can be justified by a better stress distribution due to a larger contact area between the implant and the peri-implant bone [24,25]. Moreover, increasing the implant diameter would enhance the implant primary stability. Optimal implant primary stability is affected by bone quality, implant design and diametral ratio between the hosting socket and the implant. [26,27] However, it is worth to note that the fact that a reduction in stresses on the bone is reduced by increasing the diameter of the implant does not necessarily imply that the success rate will be higher as it not only depends on bone stresses. It is necessary to take into account that the use of larger diameter implants implies the need for a greater bone volume. In this sense, Krennmair et al. did not find success rate differences among the different implant diameters evaluated in their study [28].

It should also be noted that the stress observed in the cases of angled loading was always much higher than those observed in the cases of axial loading, reaching a ninefold increase in the case of the Ø3.75 implant and Type 0-I bone quality. These results are in accordance with those reported by Sesha et al., who also observed higher bone stress when the load was applied with a 30° angulation compared to vertical loading [29]. Same findings were obtained by Papavasiliou et al., who concluded that stresses under oblique loading were approximately 10 times greater than under axial loading [30]. In its theoretical analysis done by Rangert et al. they also suggested that the axial force was more favorable and bending moments more severely stressed the implant and the bone [31].

Finally, it is important to note that calculations using finite element models represent a simplification of the real structures under study and that they only represent an approximation of the behavior of the material through 3D modeling in a virtual environment [21]. In this sense, the simplifications made in the model like, isotropic material, boundary conditions and contact behavior could affect the results. As same simplifications have been done for all the models, this allows us to do a comparative analysis between the different cases. However, it is necessary to complement this study with clinical trials to support the conclusions. 

## 5. Conclusions

An improvement in bone quality results in a reduction of induced stress in the bone. A similar effect has the increase in the diameter of the implant, although in this case the effect is much more pronounced. The stress increase when reducing the implant diameter under axial loading is much lower than that observed under oblique loading. Therefore, in cases of single-unit restorations in the molar area, it makes sense to increase the diameter of the implant, especially as the bone density decreases. Understanding the stress distribution in the bone can help the clinician to improve the choice of implant diameter depending on the anatomical factors and bone quality.

## Figures and Tables

**Table 2:** Maximum equivalent Von Mises stress (MPa) in bone at an axial load of 200N.

30°ANGLED LOAD						
Diameter (mm)	Ø3.3	Ø3.5	Ø3.75	Ø4	Ø4.25	Ø4.75
**Bone Type IV**	235	215	125	102	90	68
**Bone type III**	230	205	120	90	83	60
**Bone Type II**	205	190	110	82	71	52
**Bone Type 0-I**	180	173	97	69	58	41

**Table 3:** Maximum equivalent Von Mises stress (MPa) in bone at 30° angled load of 200N.

AXIAL LOAD						
Diameter (mm)	Ø3.3	Ø3.5	Ø3.75	Ø4	Ø4.25	Ø4.75
**Bone Type** **IV**	40	39	22	19.5	17	16
**Bone Type III**	39	38	23	19.5	17	15
**Bone Type II**	33	32	19	16	14	11
**Bone Type** **0-I**	21	20	10,5	8.5	7,5	5,5

## Data Availability

All the data obtained in this research are described in the manuscript.
